# In vivo assessment of artefacts in MRI images caused by conventional twistflex and various fixed orthodontic CAD/CAM retainers

**DOI:** 10.1007/s00056-022-00445-z

**Published:** 2023-01-26

**Authors:** Christoph J. Roser, Tim Hilgenfeld, Muhammad Abdullah Saleem, Thomas Rückschloß, Sabine Heiland, Martin Bendszus, Christopher J. Lux, Alexander Juerchott

**Affiliations:** 1 Department of Orthodontics and Dentofacial Orthopedics, Heidelberg University Hospital, Im Neuenheimer Feld 400, 69120 Heidelberg, Germany; 2 Department of Neuroradiology, Heidelberg University Hospital, Im Neuenheimer Feld 400, 69120 Heidelberg, Germany; 3 Department of Oral and Maxillofacial Surgery, Heidelberg University Hospital, Im Neuenheimer Feld 400, 69120 Heidelberg, Germany

**Keywords:** Magnetic resonance imaging, Orthodontic appliances, Computer-aided design and manufacturing, Susceptibility artifacts, Orthodontic retainers, Magnetresonanztomographie, Kieferorthopädische Apparaturen, Computergestütze Konstruktion und Fertigung, Suszeptibilitätsartefakte, Kieferorthopädische Retainer

## Abstract

**Purpose:**

To assess magnetic resonance imaging (MRI) artefacts caused by different computer-aided design/computer-aided manufacturing (CAD/CAM) retainers in comparison with conventional hand bent stainless steel twistflex retainers in vivo.

**Materials and methods:**

MRI scans (3 Tesla) were performed on a male volunteer with different CAD/CAM retainers (cobalt–chromium, CoCr; nickel–titanium, NiTi; grade 5 titanium, Ti5) and twistflex retainers inserted. A total of 126 landmarks inside and outside the retainer area (RA; from canine to canine) were evaluated by two blinded radiologists using an established five-point visibility scoring (1: excellent, 2: good, 3: moderate, 4: poor, 5: not visible). Friedman and two-tailed Wilcoxon tests were used for statistical analysis (significance level: *p* < 0.05).

**Results:**

Twistflex retainers had the strongest impact on the visibility of all landmarks inside (4.0 ± 1.5) and outside the RA (1.7 ± 1.2). In contrast, artefacts caused by CAD/CAM retainers were limited to the dental area inside the RA (CoCr: 2.2 ± 1.2) or did not impair MRI-based diagnostics in a clinically relevant way (NiTi: 1.0 ± 0.1; Ti5: 1.4 ± 0.6).

**Conclusion:**

The present study on a single test person demonstrates that conventional stainless steel twistflex retainers can severely impair the diagnostic value in head/neck and dental MRI. By contrast, CoCr CAD/CAM retainers can cause artefacts which only slightly impair dental MRI but not head/neck MRI, whereas NiTi and Ti5 CAD/CAM might be fully compatible with both head/neck and dental MRI.

## Abstract (de)

**Zusammenfassung:**

Ziel war es, den Einfluss von verschiedenen CAD/CAM(„computer-aided design/computer-aided manufacturing“)-Retainern und konventionellen handgebogenen Twistflex-Retainern auf die Entstehung von Bildartefakten in der Magnetresonanztomographie (MRT) in vivo zu untersuchen.

**Methoden:**

Bei einem männlichen Probanden wurden MRT-Aufnahmen (3 Tesla) mit verschiedenen eingesetzten CAD/CAM- (Cobalt-Chrom, CoCr; Nickel-Titan, NiTi; Titan Grad 5, Ti5) und Twistflex-Retainern durchgeführt. Insgesamt 126 Landmarken innerhalb und außerhalb des Retainerbereichs (RA; von Eckzahn zu Eckzahn) wurden von zwei Radiologen anhand eines etablierten fünfstufigen Sichtbarkeit-Scores verblindet bewertet (1 – hervorragend, 2 – gut, 3 – mäßig, 4 – schlecht, 5 – nicht sichtbar). Für die statistische Analyse wurden Friedman- und Wilcoxon-Tests verwendet (Signifikanzniveau: *p* < 0,05).

**Ergebnisse:**

Twistflex-Retainer hatten den stärksten Einfluss auf die Sichtbarkeit aller Landmarken innerhalb (4,0 ± 1,5) und außerhalb des RA (1,7 ± 1,2). Im Gegensatz dazu beschränkten sich die von CAD/CAM-Retainern verursachten Artefakte auf den Zahnbereich innerhalb des RA (CoCr: 2,2 ± 1,2) bzw. beeinträchtigten die MRT-basierte Diagnose nicht in klinisch relevanter Weise (NiTi: 1,0 ± 0,1; Ti5: 1,4 ± 0,6).

**Schlussfolgerung:**

Die vorliegende Studie an einem einzelnen Probanden zeigt, dass herkömmliche Edelstahl-Twistflex-Retainer die diagnostische Aussagekraft einer MRT-Bildgebung sowohl in der Kopf-Hals-Region als auch im Dentalbereich stark einschränken. Von CoCr-CAD/CAM-Retainern verursachte Artefakte dagegen schränken die zahnmedizinische MRT nur geringfügig ein und haben keine Auswirkung auf die MRT von Kopf und Hals. CAD/CAM-Retainer aus NiTi und Ti5 könnten sowohl für die Kopf-Hals-MRT als auch für die Dental-MRT vollständig kompatibel sein.

## Introduction

Magnetic resonance imaging (MRI) is the established non-ionizing image modality of choice for head and neck disorders [1, 2]. Moreover, MRI is also being increasingly used for dental imaging due to recent technical developments [3–7]. Several studies have demonstrated that MRI is an effective diagnostic tool in periodontology [8–12], endodontics [13–18], cariology [19], implantology [20–24], and orthodontics [25–27]. However, MRI-based diagnosis of both head/neck and dental areas can be considerably impaired by metal-induced susceptibility artefacts [28–33]. Here, orthodontic appliances are particularly relevant, as they are one of the most common causes of susceptibility artefacts in the head and neck area [34].

Studies which previously investigated orthodontic appliances for the generation of MRI artefacts mostly focused on temporarily-worn orthodontic appliances such as brackets, arches, or anchoring appliances [29–31, 33–37]. In contrast, only a small number of studies have examined MRI artefacts caused by conventional hand bent fixed retainers [30–33, 38–40]. However, these fixed retainers are especially relevant, as they are mostly worn for a lifetime [41]. Thus, not only young but also older patients who had received orthodontic therapy and now wear retainers for permanent retention can be affected by the diminished diagnostic value of MRI.

Computer-aided design/computer-aided manufacturing (CAD/CAM) retainers, which are made from materials that have not previously been used for conventional retainers, are becoming increasingly established in clinical practice. This is especially because they offer high accuracy in fit even in anatomically demanding conditions [42], show comparable results to conventional retainers with regard to their maintenance [43, 44], and might have a positive effect on oral health [45]. Importantly, CAD/CAM retainers facilitate work processes in clinical practice. In our previous in vitro pilot study, in which we investigated three CAD/CAM retainers made from different materials (nickel–titanium, NiTi; titanium grade 5, Ti5; cobalt–chromium, CoCr) and one conventional stainless-steel twistflex retainer for their influence on MRI, we identified substantial differences in artefact sizes [46]. Our previous study was intended to measure artefacts in vitro under the highest methodical standards, so as to provide the most reliable and accurate three-dimensional artefact quantification. However, drawing specific conclusions on the clinical impact of these findings was not possible due to the in vitro setting. In the present study, we investigated equivalent maxillary and mandibular CAD/CAM and twistflex retainers with regard to their impact on the visibility of both non-dental landmarks and for the first-time dental landmarks in an in vivo setting, with the aim of quantifying diagnostic impairments for head and neck MRI as well as dental MRI related to retainer-associated artefacts.

## Materials and methods

### Production of retainers

**Fig. 1:**
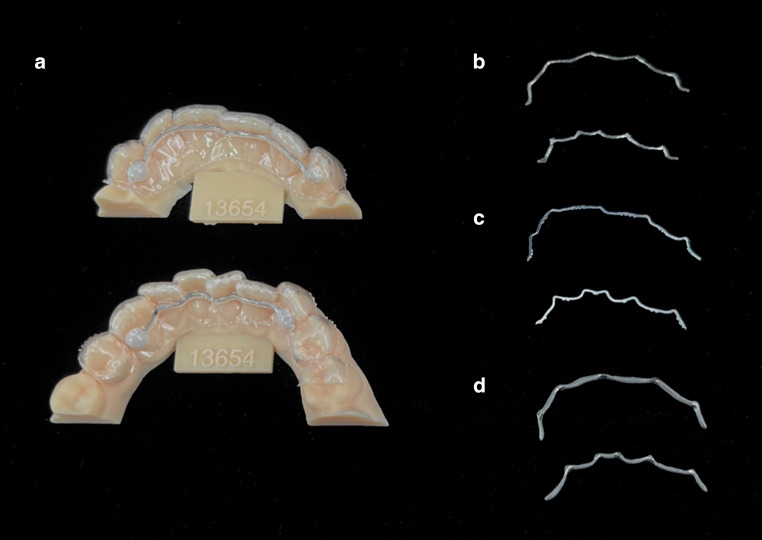
Retainers used in this study: **a** stainless-steel twistflex (hand bent) on model and embedded in acrylic splint; **b** cobalt–chromium (computer-aided design/computer-aided manufacturing [CAD/CAM]); **c** nickel–titanium (CAD/CAM); **d** grade‑5 titanium (CAD/CAM) Retainer, die in dieser Studie untersucht wurden: **a** Edelstahl-Twistflex-Retainer (handgebogen) auf dem Modell in der Schiene gefasst; **b** Cobalt-Chrom („computer-aided design/computer-aided manufacturing“ [CAD/CAM]); **c** Nickel-Titan (CAD/CAM); **d** Titan Grad 5 (CAD/CAM)

For the production of retainers, maxillary and mandibular impressions were taken with alginate (Omni Alginat, Omnident GmbH, Rodgau, Germany) from a male volunteer (aged 32) to produce plaster models from super-hard dental stone (Hinrizit, Ernst Heinrichs GmbH, Goslar, Germany). In compliance with the Declaration of Helsinki, ethical approval was obtained by the Ethics Committee of the University of University of Heidelberg (approval number: S‑452/2010) and written informed consent was obtained. The models were digitalized using a desktop scanner (Ortho X, Dentaurum, Ispringen, Germany) and the generated standard tessellation language (STL) data were sent to the respective manufacturers of CAD/CAM retainers. The twistflex retainers were produced in-house by bending on the plaster models. All relevant information on used retainers is shown in Table 1 and Fig. 1.

**Table 1:** Retainers used in this study Retainer, die in dieser Studie untersucht wurden

	Manufacturer	Product name	Manufacturing process	Material composition of retainer alloy (%)
1	Ormco (Orange, CA, USA)	Respond archwire	Bending	Stainless-steel alloy 304 (carbon: 0.08; chromium: 18/20.0; nickel: 8/10.5; magnesium: 2.0; silicon: 1.00; rest: iron)
2	Ortholize (Nienhagen, Germany)	No specific product name	CAD/CAM (laser melting)	Cobalt–chromium alloy (cobalt: 60; chromium: 28; wolfram: 9; silicon 1.5; magnesium, nitrogen, niobium, iron: all < 1)
3	Fachlabor Klee (Frankfurt, Germany)	3D Swiss Retainer	CAD/CAM (milling)	Grade‑5 titanium (aluminum: 5.5; vanadium: 3.5; iron, oxygen, nitrogen, carbon, hydrogen: all < 1; rest: titanium)
4	CA Digital (Hilden, Germany)	Memotain	CAD/CAM (laser cutting)	Nitinol (nickel: 55; titanium: 45; oxygen, nitrogen, carbon: all < 1)

*CAD/CAM* computer-aided design/computer-aided manufacturing retainer

### In vivo MRI scans and evaluation of artefact-associated clinical diagnostic impairments

For the MR measurements, all retainers were embedded into acrylic splints (Duran, Scheu Dental, Iserlohn, Germany) in accordance with previous studies [29, 33, 36]. In order to exclude potential artefact generation by the splints, MRI scans were performed with empty splints inserted prior to before the main investigation. Next, MRI was performed on a 3 T MRI system (Magnetom Tim-Trio, Siemens Healthineers, Erlangen, Germany) using a dedicated 15-channel dental coil (Mandibula, Noras MRI products GmbH, Höchberg, Germany) with maxillary and mandibular retainers inserted. For all MRI scans, a T1-weighted isotropic SPACE (sampling perfection with application optimized contrasts using different flip angle evolution) sequence was used, which has previously been shown to enable high resolution 3D MRI imaging of the craniomaxillofacial area in vivo [47]. Sequence parameters were as follows: matrix: 256 × 256; field of view: 175 mm × 175 mm; voxel size: 0.68 mm × 0.68 mm × 0.68 mm; number of sections: 192; repetition time: 800 ms; echo time: 26 ms; bandwidth: 501 Hz/pixel; slice orientation: coronal; phase-encoding direction: right-to-left; number of averages: 2; echo train length: 63; GRAPPA (generalized autocalibrating partial parallel acquisition) acceleration factor: 2; acquisition time: 6:59 min.

For quantification of artefact-related impairment of visibility, a five-point visibility score was used (1: excellent, 2: good, 3: moderate, 4: poor, 5: not visible), which was based on previously published scorings [8, 48]. In total, 126 dental and non-dental landmarks inside the retainer area (RA; i.e., from canine to canine) and outside the RA were scored independently by two blinded radiologists (each had 7 years of experience in dental MRI) using OsiriX DICOM Imaging Software (v.10.0.5, Pixmeo, Geneva, Switzerland). Before the blinded analysis of MRI scans with inserted retainers, both investigators were calibrated for landmark determination in a pilot phase using a training dataset (in vivo MRI scan without inserted retainers). After anonymization and randomization of the DICOM files, the two blinded investigators independently assessed all image datasets of the study on multiplanar reconstructions.

In order to provide detailed information concerning the visibility of different areas of interests and for statistical comparison, the landmarks were grouped as follows: (1) all landmarks inside the RA, (2) all landmarks outside the RA, (3) non-dental landmarks outside the RA, (4) dental landmarks outside the RA, (5) non-dental landmarks inside the RA, (6) dental landmarks inside the RA, (7) incisal edges inside the RA, (8) pulp chambers inside the RA, and (9) apical foramens inside the RA (Table 2).

**Table 2:** Landmarks evaluated in this study: a total of 126 dental and non-dental landmarks inside the retainer area (RA), which was from canine to canine, and outside the RA were scored for their visibility Landmarken, die in dieser Studie bewertet wurden: Insgesamt wurden 126 dentale und nicht-dentale Landmarken innerhalb des Retainerbereichs (RA; von Eck- zu Eckzahn), und außerhalb des RA hinsichtlich ihrer Sichtbarkeit analysiert

Inside the RA	*Dental (36 landmarks):* Incisal edge, pulp chamber, apical foramen of all teeth with retainer bonded (upper/lower canine to canine)
	*Non-dental (32 landmarks):* Alveolar limb of all teeth with retainer bonded (upper/lower canine to canine), anterior nasal spine, incisive foramen, subspinale point (maximum midline concavity on the maxilla), supramental point (maximum midline concavity on the mandibula), menton, anterolateral edge of the tongue right/left, tip of the tongue, anterior part of genioglossus muscle right/left, anterior part of sublingual gland right/left, anterior part of the palatal masticatory mucosa left/right, inner side of the lower lip, inner side of the upper lip, anterior part of the upper alveolar mucosa right/left, anterior part of the lower alveolar mucosa right/left
Outside the RA	*Dental (36 landmarks):* Incisal edge, pulp chamber, apical foramen from first premolar to first molar in each quadrant
	*Non-dental (20 landmarks):* Alveolar limb from first premolar to first molar in each quadrant, posterolateral edge of the tongue right/left, posterior part of the palatal masticatory mucosa left/right, posterior part of the upper alveolar mucosa left/right, and posterior part of the lower alveolar mucosa left/right, temporomandibular joint left/right

### Statistical analysis

Statistical analysis was performed using SPSS 27 (IBM, Armonk, NY, USA). First, weighted kappa (κ) values with quadratic weights and the 95% confidence interval (CI) as well as percentage agreement were calculated to determine the interrater reliability of landmark visibility scoring. We interpreted the data obtained by the scoring system as interval scaled (equal distance between the scores). Thus, we performed statistical analysis using Friedman tests to examine whether there were any statistically significant differences between the different retainers with regard to the respective groups of landmarks. Where the Friedman test revealed statically significant differences, Nemenyi post hoc tests were subsequently used for pairwise comparisons between the different retainers with regard to the respective group of landmarks. The statistical significance was set at *p* *<* 0.05.

## Results

Interrater reliability for all retainers and landmarks was substantial to almost perfect, with κ values (95% CI) of 1.000 for NiTi (1.000–1.000), 0.709 (0.552–0.867) for Ti5, 0.870 (0.804–0.936) for CoCr, and 0.956 (0.941–0.971) for twistflex retainers. The corresponding percentage agreement values were 99% (NiTi), 88% (Ti5), 84% (CoCr), and 74% (twistflex).

**Fig. 2:**
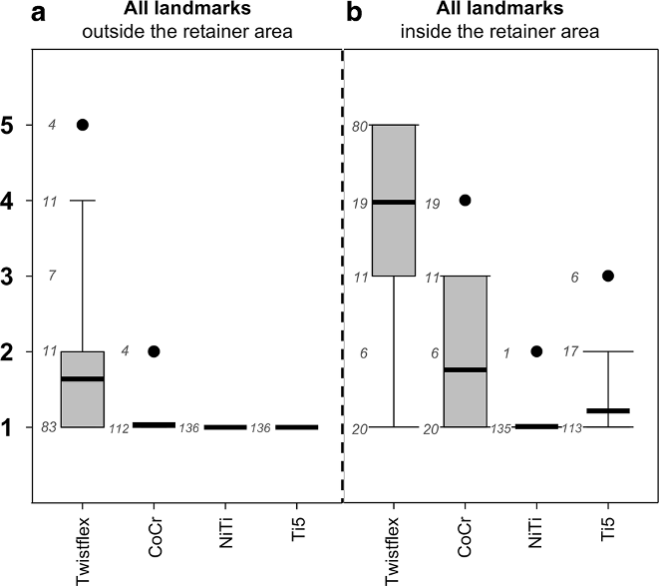
Boxplots for all landmarks outside (**a**) and inside (**b**) the retainer area (RA) with respective number of scores (*n*) next to it: Twistflex retainers were the only retainers which caused artefacts that exceeded the RA (**b**) and had the highest impact on the visibility of all landmarks inside the RA (**b**). Inside the RA, CoCr retainers had slight influence and NiTi and Ti5 retainers had no or infinitesimal influence on the visibility of all landmarks. *CoCr* cobalt–chromium, *NiTi* nickel–titanium, *Ti5* grade‑5 titanium Boxplots für alle Landmarken außerhalb (**a**) und innerhalb (**b**) des Retainerbereichs (RA) mit der entsprechenden Anzahl an Scores (*n*) daneben: Twistflex-Retainer waren die einzigen Retainer, deren Artefakte über den RA hinausgingen (**b**). Zudem hatten Twistflex-Retainer den größten Einfluss auf die Sichtbarkeit aller Landmarken innerhalb des RA (**b**). Innerhalb des RA hatten CoCr Retainer einen geringen Einfluss, NiTi- und Ti5-Retainer hatten keinen bzw. nur einen verschwindend geringen Einfluss auf die Sichtbarkeit aller Landmarken. *CoCr* Cobalt-Chrom, *NiTi* Nickel-Titan, *Ti5* Titan Grad 5

Scans with empty splints showed no artefacts. Twistflex retainers caused artefacts which had the strongest impact on the visibility of all landmarks inside the RA (visibility score [VS] ± standard deviation; 4.0 ± 1.5) and were the only retainers that resulted in systematic impairment of landmarks’ visibility outside the RA (1.6 ± 1.2; Fig. 2).

**Fig. 3:**
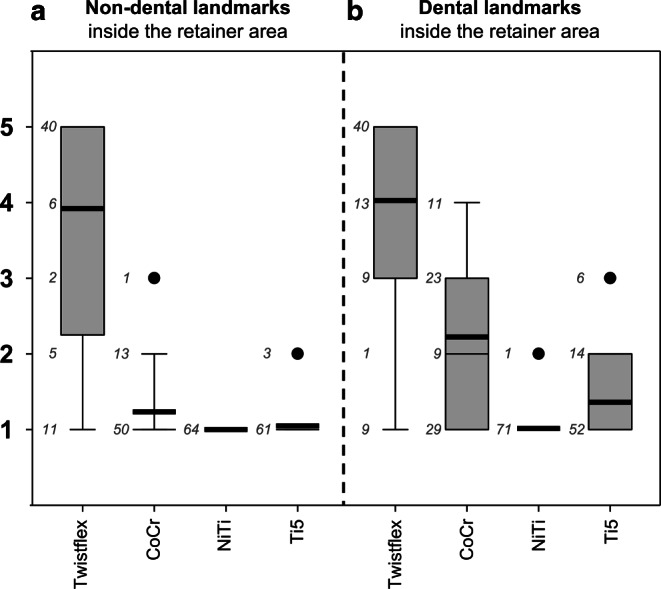
Boxplots for non-dental and dental landmarks inside the retainer area (RA) with respective number of scores (*n*) next to it: In contrast to twistflex retainers, all computer-aided design/computer-aided manufacturing (CAD/CAM) retainers had no or infinitesimal influence on the visibility of non-dental landmarks inside the RA (**a**) but differed with regard to their influence on the visibility of dental landmarks inside the RA (**b**). *CoCr* cobalt–chromium, *NiTi* nickel–titanium, *Ti5* grade‑5 titanium Boxplots für nichtdentale und dentale Landmarken im Retainerbereich (RA) mit der jeweiligen Anzahl an Scores (*n*) daneben: Im Gegensatz zu Twistflex-Retainern hatten alle CAD/CAM(„computer-aided design/computer-aided manufacturing“)-Retainer keinen oder nur verschwindend geringen Einfluss auf die Sichtbarkeit nichtdentaler Landmarken innerhalb des RA (**a**), unterschieden sich jedoch hinsichtlich des Einflusses auf die Sichtbarkeit dentaler Landmarken innerhalb des RA (**b**). *CoCr* Cobalt-Chrom, *NiTi* Nickel-Titan, *Ti5* Titan Grad 5

Inside the RA, twistflex retainers severely impaired the visibility of dental landmarks (overall score: 4.0 ± 1.3; incisal edges: 5.0 ± 0.0, pulp chambers: 4.5 ± 0.7, apical foramens: 2.6 ± 1.4; Fig. 3) and non-dental landmarks (3.9 ± 1.6; 4). Non-dental landmarks whose visibility was severely limited (score ≥ 4) by twistflex retainers included right and left anterolateral edge of the tongue, tip of the tongue, anterior part of the palatal masticatory mucosa, inner side of the lower and upper lip, and anterior part of the lower and upper alveolar mucosa.

**Fig. 4:**
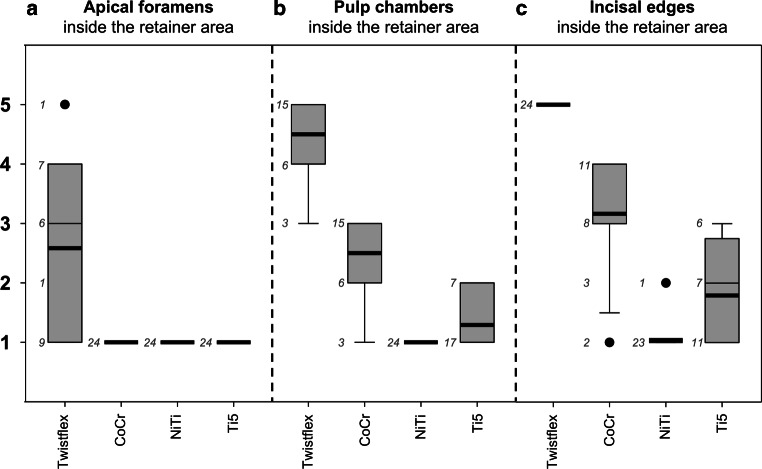
Boxplots for apical foramens, pulp chambers and incisal edges in the retainer area (RA) with respective number of scores (*n*) next to it: Only twistflex retainers had an influence on the visibility of apical foramens inside the RA (**a**). Within computer-aided design/computer-aided manufacturing (CAD/CAM) retainers, CoCr retainers had a moderate influence on the visibility of the pulp chambers (**b**) and the incisal edges (**c**). In contrast, NiTi and Ti5 retainers had no or minimal influence on the visibility on the respective dental landmarks. *CoCr* cobalt–chromium, *NiTi* nickel–titanium, *Ti5* grade‑5 titanium Boxplots für apikale Foramina, Pulpenkammern und Inzisalkanten im Retainerbereich (RA) mit der jeweiligen Anzahl an Scores (*n*) daneben: Ausschließlich Twistflex-Retainer hatten einen Einfluss auf die Sichtbarkeit der apikalen Foramina innerhalb des RA (**a**). Unter den CAD/CAM(„computer-aided design/computer-aided manufacturing“)-Retainern hatten CoCr-Retainer einen moderaten Einfluss auf die Sichtbarkeit der Pulpakammern (**b**) und Inzisalkanten (**c**). NiTi- und Ti5-Retainer dagegen hatten keinen oder nur minimalen Einfluss auf die Sichtbarkeit der jeweiligen dentalen Landmarken. *CoCr* Cobalt-Chrom, *NiTi* Nickel-Titan, *Ti5* Titan Grad 5

**Fig. 5:**
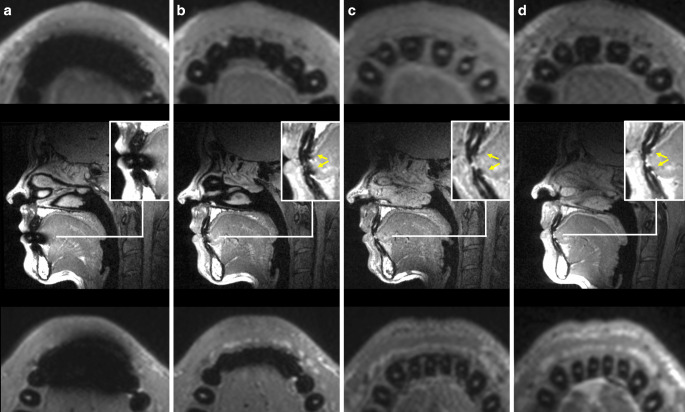
MRI images: Transversal plane of upper jaw (*top*), sagittal plane (*middle*), transversal plane of lower jaw (*bottom*); artefacts for CoCr (**b**), NiTi (**c**) and Ti5 (**d**) retainers marked with *yellow arrows*—twistflex retainers caused artefacts which impaired the visibility of both non-dental and dental landmarks (**a**). CoCr retainers impaired the visibility of the pulp and the incisal edges (**b**). NiTi (**c**) and Ti5 retainers (**d**) had no impact on the visibility of both non-dental and dental landmarks. *CoCr* cobalt–chromium, *NiTi* nickel–titanium, *Ti5* grade‑5 titanium MRT-Aufnahmen: Transversale Ebene des Oberkiefers (*oben*), sagittale Ebene (*Mitte*), transversale Ebene des Unterkiefers (*unten*); Artefakte von CoCr- (**b**), NiTi- (**c**), Ti5- (**d**) Retainern mit *gelben Pfeilen* markiert – Twistflex-Retainer verursachten als einzige Retainer Artefakte, welche die Sichtbarkeit von dentalen und nichtdentalen Landmarken beeinträchtigten (**a**). CoCr-Retainer beeinträchtigten die Sichtbarkeit der Pulpae und Inzisalkanten (**b**). NiTi- (**c**) und Ti5-Retainer (**d**) hatten keinen Einfluss auf die Sichtbarkeit dentaler und nichtdentaler Landmarken. *CoCr* Cobalt-Chrom, *NiTi* Nickel-Titan, *Ti5* Titan Grad 5

In contrast, none of the CAD/CAM retainers caused artefacts which exceeded the RA. Moreover, all CAD/CAM retainers had significantly less influence on the visibility on both dental and non-dental landmarks inside the RA (each *p* < 0.01) compared to twistflex retainers. Regarding the dental landmarks inside the RA, CoCr retainers had moderate influence on the visibility (overall score: 2.2 ± 1.2; incisal edges: 3.2 ± 1.0, pulp chambers: 2.5 ± 0.7, apical foramens: 1.0 ± 0.0). In contrast, both NiTi (overall score: 1.0 ± 0.1; incisal edges: 1.0 ± 0.2, pulp chambers: 1.0 ± 0.0, apical foramens: 1.0 ± 0.0) and Ti5 retainers (overall score: 1.4 ± 0.6; incisal edges: 1.8 ± 0.8; pulp chambers: 1.3 ± 0.5; apical foramens: 1.0 ± 0.0) had no or only minimal impact on visibility. With regard to non-dental landmarks inside the RA, CoCr retainers (1.2 ± 0.5) only had infinitesimal influence and both NiTi (1.0 ± 0) and Ti5 (1.0 ± 0.2) CAD/CAM retainers had no influence on visibility. The mean results of the respective groups of landmarks are shown in Figs. 2, 3 and 4. Moreover, the respective MRI images are shown in Fig. 5.

## Discussion

The present study demonstrated that MRI artefacts produced by CAD/CAM retainers had only minor (CoCr) or no impairment (NiTi and Ti5) on the diagnostic value of head and neck MRI as well as dental MRI in vivo. However, conventional stainless steel twistflex retainers have the potential to cause severe diagnostic impairment in the craniofacial area, completely or substantially diminishing the diagnostic value of dental MRI/head and neck MRI. These results are of high clinical significance due to the increasing number of patients with fixed retainers who are referred for MRI scans and due to the growing importance of CAD/CAM retainers in clinical practice [49–51]. Therefore, we propose that MRI characteristics of retainers should be taken into consideration when choosing retainer materials, at least in certain cases.

In our previous in vitro study, we used a highly standardized method for artefact quantification but were not able to draw specific conclusions regarding diagnostic impairment in a clinical context [46]. In the present study, we used the same high-resolution 3D isotropic SPACE sequence in vivo to assess retainer artefacts under clinical conditions. The applied SPACE sequence was previously shown to be ideal for the high-resolution 3D MRI imaging of the craniomaxillofacial area [25]. With a scanning time of 7 min, the sequence is applicable in clinical routine MRI imaging. Using this sequence enabled the detailed multiplanar analysis of artefacts and therefore the assessment of multiple small anatomical structures that were directly adjacent to the retainers, including dental MRI-related landmarks. This stands in contrast to most previous in vivo studies that used conventional MRI sequences with anisotropic voxels and/or lower spatial resolution [30–33, 37, 38].

Comparison of the results from the present study with previous findings is limited because previous studies mostly focused on temporarily worn orthodontic appliances [29, 34, 36, 52]. However, fixed retainers are especially important because they remain in place throughout one’s life and therefore MRI involving inserted retainers is common [41]. Nevertheless, only a few in vivo studies or studies on a human skull investigated conventional fixed retainers for their influence on the visibility of different non-dental landmarks. Importantly, only one or two retainers were included in these previous studies [30–33, 37, 38, 40]. To the best of our knowledge, no study has previously investigated CAD/CAM retainers for their influence on MRI; moreover, there was no prior in vivo study which investigated Ti5 and CoCr retainers in general. Therefore, the results of the present study are of high clinical relevance, not only because CAD/CAM retainers have become established in dental practice due to rapidly developing CAD/CAM technologies but also because they are made from materials that were not previously used in the production of conventional retainers.

The results of the present study demonstrated that stainless steel twistflex retainers generated artefacts which severely impaired the visibility of non-dental landmarks. Moreover, they were the only retainers in the present study that caused artefacts that exceeded the RA. These results stand in line with previous investigations, which all found severe MRI artefacts to be associated with stainless steel retainers [30–33, 40]. Artefacts caused by twistflex retainers are of particular clinical relevance as these retainers are widely used because of their proven long-term success [53]. However, for MR imaging as a modality of choice in the head/neck region, stainless steel twistflex retainer-induced artefacts may severely impact the assessment of adjacent tissues (e.g., the palatal mucosa, the alveolar mucosa, the lips or the anterolateral edge, and tip of the tongue). This is particularly relevant for the diagnosis of oral cancer, especially of the anterior tongue, which is increasingly affecting younger patients [54, 55].

None of the CAD/CAM retainers severely impaired the diagnosis of non-dental structures. Only CoCr retainers caused artefacts which had a slight influence on the visibility of the anterolateral edge and tip of the tongue. However, all landmarks were still of sufficient visibility (score ≤ 2) when the CoCr retainers were inserted. But CAD/CAM retainers differed with regard to their influence on the visibility of dental landmarks. NiTi and Ti5 retainers showed almost no artefacts and therefore did not impact the visibility of dental landmarks, which for NiTi stands in line with a previous study on conventional hand bent NiTi retainers [30]. In contrast, CoCr retainers revealed artefacts that impaired the visibility of the dental crown and slightly impaired the visibility of the pulp chamber. This might have a potential impact in the future because of the increasing application of MRI in dentistry. In particular, patients wearing CoCr retainers might not benefit from the increasing use of MRI-based evaluation of endodontic structures [18]. Moreover, MRI-based caries diagnosis [19] and implementations, which require accurate visualization of the incisal edges such as through MRI-based implantology planning [20], might not be possible when CoCr retainers are inserted. By contrast, patients wearing NiTi and Ti5 retainers might benefit from unrestricted diagnosis of both head/neck and dental imaging and may therefore be protected from unnecessary repetition of artefact scans leading to delayed diagnosis. Moreover, wearing these retainers might prevent the unnecessary removal of the artefact-prone retainers prior to MRI imaging and therefore unnecessary detrimental effects such as enamel damage, expense, or an orthodontic relapse [41, 56].

Several limitations have to be considered when interpreting the results of the present study. We used an optimized T1-weighted sequence for artefact measurements because this simulates conditions which can be compared to a standard clinical situation. Several variables such as different field strength and sequences could also affect extent of MRI artefacts [57]. Furthermore, it is important to mention that we used only one test person for our examination in order to place the results of our previous in vitro study [46] in a clinical context. However, differences in anatomy between patients might have an impact on the visibility of landmarks, which are located directly adjacent to the retainer. Therefore, further in vivo studies with larger sample sizes should be conducted, also as in particular CAD/CAM technology involving additional manufacturing materials will continue to develop.

## Conclusions

The unfavorable effects of retainer-associated artefacts on the diagnostic value of dental magnetic resonance imaging (MRI) and head/neck MRI are of major clinical relevance for both orthodontists and radiologists. The results of this study provide an important basis for deciding on the retainer material. Within the limitations of our study, the following conclusions can be drawn:

- Conventional stainless steel twistflex retainers can generate artefacts which completely/substantially impair the diagnostic value of dental MRI/head and neck MRI.
- CoCr CAD/CAM retainers can cause artefacts which slightly impair dental MRI but not head and neck MRI.
- NiTi and Ti5 CAD/CAM retainers might not impair either dental or head and neck MRI.

As MRI represents a routine imaging modality and retainers are often worn lifelong, we suggest that the MRI characteristics of the material should be considered by orthodontists.
